# Safety and effectiveness of post percutaneous coronary intervention physiological assessment: Retrospective data from the post-revascularization optimization and physiological evaluation of intermediate lesions using fractional flow reserve registry

**DOI:** 10.3389/fcvm.2022.983003

**Published:** 2022-08-18

**Authors:** Antonio Maria Leone, Stefano Migliaro, Giuseppe Zimbardo, Pio Cialdella, Eloisa Basile, Domenico Galante, Federico Di Giusto, Gianluca Anastasia, Andrea Vicere, Edoardo Petrolati, Antonio Di Stefano, Giorgia Campaniello, Domenico D’Amario, Rocco Vergallo, Rocco Antonio Montone, Antonino Buffon, Enrico Romagnoli, Cristina Aurigemma, Francesco Burzotta, Carlo Trani, Filippo Crea

**Affiliations:** ^1^Dipartimento di Scienze Cardiovascolari, Fondazione Policlinico Universitario Agostino Gemelli (IRCCS), Rome, Italy; ^2^Dipartimento di Scienze Cardiovascolari, Università Cattolica del Sacro Cuore, Rome, Italy; ^3^Policlinico Casilino, Rome, Italy

**Keywords:** FFR, PCI, physiology-guided optimization, post-PCI evaluation, function based management CCS (chronic coronary syndrome)

## Abstract

**Background:**

While the importance of invasive physiological assessment (IPA) to choose coronary lesions to be treated is ascertained, its role after PCI is less established. We evaluated feasibility and efficacy of Physiology-guided PCI in the everyday practice in a retrospective registry performed in a single high-volume and “physiology-believer” center.

**Materials and methods:**

The PROPHET-FFR study (NCT05056662) patients undergoing an IPA in 2015–2020 were retrospectively enrolled in three groups: Control group comprising patients for whom PCI was deferred based on a IPA; Angiography-Guided PCI group comprising patients undergoing PCI based on an IPA but without a post-PCI IPA; Physiology-guided PCI group comprising patients undergoing PCI based on an IPA and an IPA after PCI, followed by a physiology-guided optimization, if indicated. Optimal result was defined by an FFR value ≥ 0.90.

**Results:**

A total of 1,322 patients with 1,591 lesions were available for the analysis. 893 patients (67.5%) in Control Group, 249 patients (18.8%) in Angiography-guided PCI Group and 180 patients (13.6%) in Physiology-guided PCI group. In 89 patients a suboptimal functional result was achieved that was optimized in 22 cases leading to a “Final FFR” value of 0.90 ± 0.04 in Angiography-Guided PCI group. Procedural time, costs, and rate of complications were similar. At follow up the rate of MACEs for the Physiology-guided PCI group was similar to the Control Group (7.2% vs. 8.2%, *p* = 0.765) and significantly lower than the Angiography-guided PCI Group (14.9%, *p* < 0.001), mainly driven by a reduction in TVRs.

**Conclusion:**

“Physiology-guided PCI” is a feasible strategy with a favorable impact on mid-term prognosis. Prospective studies using a standardized IPA are warrant to confirm these data.

## Background

While the importance of invasive physiological assessment (IPA) to choose coronary lesions to be treated is ascertained, its role in evaluating the immediate result of PCI and its optimization is much less established (1, 2). Nevertheless, the unmet need for a help in assessing result of revascularization is evident when considering that one in four patients after an angiographically successful PCI still experiences recurrent angina, persistence of documented ischemia or MACEs within 2 years (3–6). Given the excellent performance of invasive physiological assessment evaluation for diagnostic purposes, it seems intuitive to utilize this approach also to assess PCI result. Yet, the association between post-PCI fractional flow reserve (FFR) results and risk of subsequent events is still debated (7).

The aim of this study was to evaluate the use of physiology-guided PCI in terms of prevalence, feasibility, and performance on in-hospital and mid-term outcomes in contemporary interventional procedures from the Cath Lab performing the largest number of IPAs in Italy.^1^

## Materials and methods

### Study design and population

**FIGURE 1:**
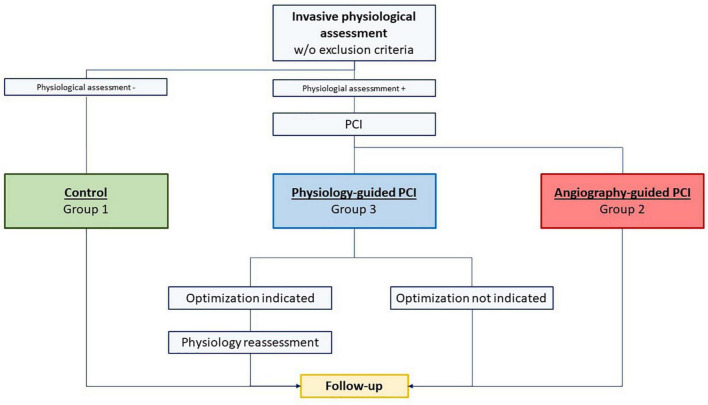
Study flow chart: group 1 comprised patients for whom percutaneous coronary intervention (PCI) was deferred based on an invasive physiological assessment [Fractional flow reserve (FFR) > 0.80 and/or non-hyperemic pressure ratio (NHPR) > 0.89 and/or contrast FFR (cFFR) > 0.87] (Control Group); group 2 comprised patients who underwent PCI based on an invasive physiological assessment but without any subsequent invasive physiological assessment after PCI (Angiography-Guided PCI); group 3 comprised patients who underwent PCI based on an invasive physiological assessment and with a subsequent invasive physiological assessment after PCI (Physiology-guided PCI) followed by a physiology-guided optimization, if indicated.

The Post-Revascularization Optimization and Physiological Evaluation of intermediate Lesions using FFR (PROPHET-FFR) is a single center ambispective study held at Policlinico Universitario A. Gemelli IRCCS in Rome evaluating the feasibility and the technical and clinical efficacy of Physiology-guided PCI. All patients able to give a valid informed consent and undergoing an IPA at the Fondazione Policlinico Universitario A. Gemelli IRCCS in Rome (Italy) are enrolled and included in one of the following three groups as described in Figure 1:

1. Group 1 comprising patients for whom PCI is deferred based on an IPA [FFR > 0.80 and/or non-hyperaemic pressure ratio (NHPR) > 0.89 and/or contrast FFR (cFFR) > 0.87) (Control Group).
2. Group 2 comprising patients who undergo PCI based on an IPA but without any subsequent IPA after PCI (Angiography-Guided PCI).
3. Group 3 comprising patients who undergo PCI based on an IPA and with a subsequent IPA after PCI (Physiology-guided PCI) followed by a physiology-guided optimization, if indicated.

Considering the ambispective nature of the study, the PROPHET-FFR study is divided in a first retrospective phase followed by a second prospective phase. In the former, the operators were free to decide the strategy. The latter is currently ongoing and the operators are again free to decide the strategy but in case of choice of Physiology-guided PCI they are recommended to adhere to the flow chart described in our recent review paper (8). We report here the results of the retrospective phase.

All patients with acute and chronic coronary syndromes referred to our center from January 2015 to June 2020 for physiological assessment of an angiographically intermediate coronary stenosis, not presenting any of the exclusion criteria below reported were retrospectively enrolled. For ST segment elevation myocardial infarction (STEMI) non-culprit lesions only were enrolled. Exclusion criteria were the following: history of severe poorly uncontrolled pulmonary disease, hemodynamic instability during the diagnostic or therapeutic procedure, need of mechanical circulatory or ventilatory support, life expectancy < 1-year, indication to surgical revascularization, major procedural complications during percutaneous revascularization making post-PCI functional evaluation unsafe or impossible (cardiac arrest needing cardiopulmonary resuscitation, major bleeding, large iatrogenic coronary dissection, coronary embolization in a main vessel, suspected stroke, coronary no-reflow).

The protocol was approved by the Ethics Committee of the Fondazione Policlinico Universitario A. Gemelli IRCCS of Rome and is registered in Clinicaltrials.gov (NCT05056662).

### Study protocol

After coronary angiography, baseline IPA was performed in every patient. Because of the observational nature of the study, the choice about the index to adopt [either NHPRs including instantaneous wave free ratio (iFR), resting full-cycle ratio (RFR), and diastolic resting pressure ratio (dPR)], conventional FFR or contrast-FFR (cFFR) and its interpretation were left to operators’ discretion. Physiological assessments were performed using Pressure Wire Certus and Pressure Wire X by Abbott Vascular Inc., Verrata Plus by Philips Inc., and Navvus catheter by Acist Inc.

For FFR assessment, hyperemia was obtained using adenosine, administered either by intra-venous (9) or intra-coronary route (10). The operators were encouraged to place the wire as distal as possible in the vessel and decided about indication and modality of revascularization according to the physiological results. We excluded only those patients treated with PCI despite the evidence of non-ischemic values for all indexes.

At the end of PCI operators were free to choose if and how to repeat IPA and to decide about the opportunity and modality of performing an additional optimization with an optional final physiological assessment. Similarly, the choice of performing a pullback maneuver prior and after PCI was left to operator’s discretion.

“Post-PCI” IPA was defined as the assessment performed once the operator considered the revascularization completed based on the angiographic assessment. “Post-optimization” IPA was considered the assessment made after any further action taken to improve the physiological result. Consequently, “Final results” comprised both “Post PCI results,” for those cases not further optimized and “Post optimization results” in case of optimization and retesting.

### Data collection, follow-up, and study endpoints

All baseline demographic, clinical, and procedural data were retrieved from the patient database of Fondazione Policlinico Universitario A. Gemelli IRCCS, Rome. Clinical follow-up was performed by telephone interviews and out-patient visits performed according to a pre-specified set of questions by a group of physicians unaware of the details of the procedure and consequently of the grouping. Clinical data were integrated with hospital records in case of need. Follow up was censored at 3 years from index procedure. Events were adjudicated by two independent operators (SM and PC) and a random cluster review, blinded to group affiliation, was periodically performed by the Principal Investigator (AL).

Primary endpoint of the study was the first occurrence of a major adverse cardiovascular event (MACE) defined as the composite of cardiac death (CD), spontaneous myocardial infarction (MI), and target vessel revascularization (TVR). CD was defined according to Academic Research Consortium-2 indication (11), MI was defined according to ESC 4th definition of Myocardial Infarction (12), TVR was defined as the clinically driven revascularization of the target vessel. Secondary endpoints were the single components of MACE as well as the rate of unplanned hospitalizations leading to coronary angiography.

In addition, procedural and in-hospital outcome was also investigated by analyzing radioscopic time, amount of contrast medium, post-procedural release of markers of myonecrosis, rate of peri-procedural myocardial injury and of type 4a MI and global costs associated with the different strategies according to our previously published paper (13).

### Study groups and statistical analysis

The study flow chart is presented in Figure 1. As previously mentioned, patients were divided into three groups: Group 1 (Control Group), Group 2 (Angiography-guided PCI), and Group 3 (Physiology-guided PCI). Comparisons were made among the three groups.

Categorical variables were reported as frequencies and percentages while continuous variables were reported as means with standard deviation or median with interquartile range according to normality. Differences among categorical variables were assessed using Pearson χ^2^ test while differences among continuous variables were calculated using the independent ANOVA test. Differences among medians were compared by Mann–Whitney test. *Post hoc* analysis with Bonferroni’s correction was used to identify pairwise differences. Survival was analyzed by Kaplan Meier method with stratified log-rank test to show the incidence of clinical endpoints and to check for intergroup differences and with Cox proportional hazard ratio method to identify the impact in time of clinical variables of interest. ROC curve analysis was run to identify the best cut-off value of final FFR to predict the outcomes. All tests were 2-tailed, and a *P* < 0.05 was considered statistically significant. Statistical analyses were performed using SPSS statistics for Windows, version 24.0 (SPSS, Chicago, IL, IBM Inc., United States) and GraphPad 9.0 (GraphPad Software, San Diego, CA, United States).

## Results

### Baseline characteristics

A total of 1,556 patients were screened for enrolment: 54 patients denied informed consent, 47 were managed at variance with the initial IPA, and 133 were lost to follow up. Consequently, a total of 1,322 patients with 1,591 lesions were available for the analysis. Among these, 893 patients (67.5%) with 1,137 lesions (71.4%) were included in Group 1 (Control Group), 249 patients (18.8%) with 265 lesions (16.6%) in Group 2 (Angiography-guided PCI), and 180 patients (13.6%) with 189 lesions (11.8%) in Group 3 (Physiology-guided PCI). In three cases a loss of connection or a damage of the pressure wire was observed and in two cases the operator chose to abandon rewiring of the stented segment after a gentle attempt. In all cases the operator had the opportunity to open a new device for the physiological assessment or to use a more aggressive approach but chose to avoid it based on the good angiographic result. These cases were included in Group 2.

Baseline clinical characteristics of the groups are reported in Tables 1, 2.

**TABLE 1:** Clinical characteristics of the patients’ groups.

Patients (*n* = 1,322)	Group 1 (*n* = 893)	Group 2 (*n* = 249)	Group 3 (*n* = 180)	*P*-value (global)
Age (years)	69.2 ± 10.6	69.1 ± 9.7	68.1 ± 10.0	0.480
BMI	26.7 ± 4.4	26.1 ± 3.7	27.5 ± 5.0	0.108
Male gender	617 (69.1)	193 (77.5)	146 (81.5)	0.001
Hypertension	742 (83.6)	214 (85.9)	146 (81.6)	0.465
EF	57.1 ± 8.9	57.4 ± 8.7	57.3 ± 9.1	0.908
Ejection fraction < 40%	39 (4.4)	12 (4.8)	7 (3.9)	0.897
Dyslipidemia	558 (62.8)	164 (66.1)	113 (62.8)	0.623
Statin	512 (61.1)	158 (66.1)	105 (66.0)	0.239
Diabetes	234 (26.5)	70 (28.1)	69 (38.3)	0.006
Smoke	402 (46.2)	123 (50.6)	82 (49.1)	0.428
Beta blockers	590 (66)	168 (67.4)	111 (61.6)	0.539
ACE-I	579 (68.1)	179 (71.8)	109 (60.5)	0.201
CCB	146 (16.3)	43 (17.2)	25 (13.8)	0.833
DIURETICS	159 (17.8)	59 (22.4)	24 (13.3)	0.107
CKD	67 (7.5)	22 (8.8)	18 (10)	0.650
MDRD female	76.7 ± 29.3	72.6 ± 31.6	84.5 ± 36.6	0.474
MDRD male	79.4 ± 28.5	84.2 ± 22.6	81.6 ± 23.1	0.411
Family history	244 (27.3)	61 (24.5)	48 (26.6)	0.626
Previous MI	198 (22.2)	66 (26.5)	40 (22.2)	0.344
Previous PCI	319 (35.7)	92 (36.9)	56 (31.1)	0.769
Previous CABG	27 (3.0)	8 (3.2)	6 (3.3)	0.970
Clinical presentation				< 0.001
CCS	669 (74.9)	161 (64.7)	139 (77.2)	
NSTEACS	158 (17.7)	77 (30.9)	33 (18.3)	
STEACS	66 (7.4)	11 (4.4)	8 (4.4)	
Multivessel disease				< 0.001
2VD	88 (9.8)	75 (30.1)	50 (27.7)	
3VD	27 (3.0)	22 (8.8)	15 (8.3)	
PCI on other vessel	207 (23.2)	74 (29.7)	56 (31.1)	0.020
LAD involvement	638 (71.40)	218 (87.5)	147 (81.6)	< 0.001

**TABLE 2:** Procedural characteristics of the lesions’ groups.

Lesions (*n* = 1,591)	Group 1 (*n* = 1,137)	Group 2 (*n* = 265)	Group 3 (*n* = 189)	*p*-value (global)	*p*-value (3 vs. 2)
In stent lesion	51 (4.5)	12 (4.5)	15 (7.9)	0.120	
Lesion site				< 0.001	
LAD	743 (65.3)	227 (83.7)	160 (84.6)		
Cx	228 (20.1)	13 (4.9)	11 (5.8)		
RCA	166 (14.6)	25 (9.4)	18 (9.5)		
Hyperemia induction				0.985	
Adenosine ic	248 (24.2)	56 (24.1)	37 (23.6)		
Adenosine iv	777 (75.8)	176 (75.9)	120 (76.4)		
Angiographic lesion severity	53.4 ± 9.1	62.1 ± 9.9	64.4 ± 11.5	0.462	
Stent length		33.4 ± 17.6	34.7 ± 17.0		0.472
Number of stent, median (IQR)		1 (1–2)	1 (1–2)		0.354
Number stent, average		1.3 ± 0.60	1.27 ± 0.64		0.241
Stent diameter, mm		3.0 ± 0.42	2.95 ± 0.34		0.203
Optimization by post-dilation, n (%)			10 (5.3)		
Optimization with a new stent, n (%)			12 (6.3)		
**Physiological parameters**					
Baseline Pd/Pa	0.95 ± 0.03	0.89 ± 0.05	0.89 ± 0.05	< 0.001	1
Baseline FFR	0.88 ± 0.04	0.76 ± 0.03	0.76 ± 0.03	< 0.001	0.082
Baseline IFR	0.92 ± 0.05	0.82 ± 0.08	0.87 ± 0.03	< 0.001	1.0
Baseline cFFR	0.91 ± 0.06	0.81 ± 0.03	0.80 ± 0.05	< 0.001	1.0
Post-PCI Pd/Pa			0.94 ± 0.03		
Post-PCI FFR			0.89 ± 0.05		
Post-PCI IFR			0.92 ± 0.03		
Post-PCI cFFR			0.89 ± 0.04		
Post-optimization Pd/Pa			0.94 ± 0.02		
Post-optimization cFFR			0.91 ± 0.03		
Post-optimization FFR			0.92 ± 0.05		
Final FFR			0.90 ± 0.04		
Final cFFR			0.90 ± 0.03		

Mean age was 69.1 ± 10.4 years. Overall, most of patients were male (72.3%) but males were slightly less represented in Group 1 vs. the others [617 (69.1%) vs. 193 (77.5%) vs. 146 (81.5%), *p* < 0.001]. Interestingly, diabetic patients were significantly more prevalent in group 3 vs. the others [69 (38.3%) vs. 234 (26.5%) and 70 (28.1%), *p* = 0.006)]. Mean left ventricular ejection fraction was 57.2% ± 8.9% while 58 patients (4.3%) only had a left ventricular ejection fraction < 40% with a similar distribution among groups. Admission diagnosis was chronic coronary syndrome (CCS) in 73.3% of cases, non-ST segment elevation acute coronary syndromes (NSTEACS) in 20.2% and ST segment elevation myocardial infarction (STEMI) in 6.4%. In Group 2 a higher prevalence of NSTEACS at the cost of less CCS presentations was observed [NSTEACS: 158 (17.7%) vs. 77 (30.9%) vs. 33 (18.3%), *p* < 0.001; CCS 669 (74.9%) vs. 161 (64.7%) vs. 139 (77.2%), *p* < 0.001)]. More than one third of the patients experienced a previous PCI while almost one in four patients had a previous myocardial infarction with no difference among groups. The majority of the lesions were located in the left anterior descending (LAD) artery, but non-LAD lesions were more frequent in Group 1 which also had a lower incidence of multivessel disease.

Final diagnostic decision was driven by conventional FFR in 1,193 (74.9%) lesions, by cFFR alone in 280 (17.5%) lesions and by NHPRs in 104 (RFR/iFR 62 + Pd/Pa 42) (6.5%) lesions.

As expected, mean baseline FFR was significantly higher in Group 1 (0.88 ± 0.04) in comparison to both other groups that, conversely, did not differ from each other (0.76 ± 0.03 vs. 0.76 ± 0.04).

### “Post-PCI” invasive physiological assessment

**FIGURE 2:**
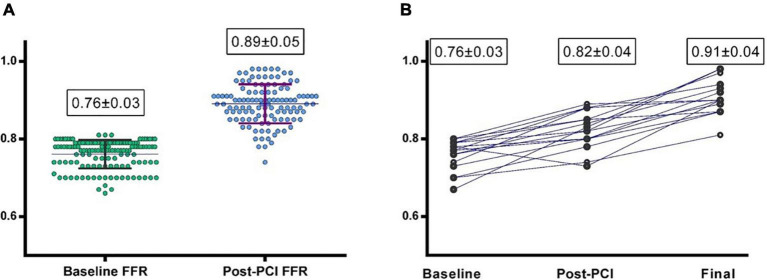
**(A)** Baseline and post-PCI fractional flow reserve (FFR) values in group 3 (Physiology-guided PCI). **(B)** Baseline, post PCI and final FFR values in the 22 patients undergoing “Physiology-guided Optimization.”

From the 180 patients of Group 3, “Post-PCI” IPA was performed by conventional FFR in 114 (60.3%) cases, by cFFR in 62 (32.8%), and by NHPRs in 13 (7 iFR/RFR + 6 Pd/Pa) (6.8%) cases. The mean “post PCI” FFR was 0.89 ± 0.05 with an average increase after PCI (and before any optimization) of 19 ± 8.5% (Figure 2A). In 58 (30.6%) lesions post-PCI FFR value was < 0.90 while only in 4 (2.1%) cases was < 0.80. In those patients in which a non-FFR based approach was used, 28 (14.8%) lesions showed a cFFR < 0.90, 1 (0.5%) a iFR/RFR < 0.90 and 2 (1%) Pd/Pa < 0.92.

A total of 22 (11.6%) lesions underwent physiology-guided optimization; in 12 of these cases the optimization was driven by a post-PCI FFR value < 0.90 and in 8 of these cases by cFFR < 0.90. Optimization consisted of post-dilation of the previously implanted stent with a larger balloon in 10 cases and of the implantation of another stent proximally or distally in 4 and 6 cases, respectively.

“Post-optimization” result was reassessed by FFR in nine of these cases, by cFFR in 10 cases, while the remaining were retested by NHPRs; of these optimized lesions, only two were left with an FFR post-optimization < 0.90 and only three cases ended with cFFR < 0.90.

The mean “Post-optimization” FFR value was 0.92 ± 0.05 with an FFR improvement from baseline of 18% ± 7.4%. This led to a mean overall “Final FFR” value of 0.90 ± 0.04 for Group 3 (Figure 2B; Table 2) that was even significantly higher than FFR in Group 1 (*p* < 0.001).

### Procedural and in-hospital outcomes

Procedural and in-hospital outcomes are presented in Table 3. Procedural time was higher in Group 2 and 3 (19.8 ± 12.2 and 21.6 ± 8.9 min) than in Group 1 (12.7 ± 9.5, p < 0.001) but without a significant difference among them (*p* = 0.56). Physiology-guided PCI required a higher amount of contrast medium compared to Angiography-guided PCI and Control Group (278.7 ± 168.4 ml vs. 163.0 ± 69.0 and 236.3 ± 116.6, *p* < 0.001) while no difference in post-procedural creatinine and post-procedural release of myocardial damage markers was observed. While Physiology-guided PCI did not increase the overall costs compared to Angio-guided PCI (3152 ± 1814 vs. 3329 ± 2325 euros, *p* = 1), it was associated to a reduced length of stay (6.0 ± 4.3 vs. 8.5 ± 6.4 days, *p* < 0.001). No significant difference was seen for the number, total length and mean diameter of implanted stents between Groups 2 and 3 (Table 2) as well for the use of intravascular imaging (Table 4).

**TABLE 3:** In-hospital outcomes.

Patients (*n* = 1,322)	Group 1 (*n* = 893)	Group 2 (*n* = 249)	Group 3 (*n* = 180)	*p*-value	*p*-value (3 vs. 2)	*p*-value (3 vs. 1)	*p*-value (2 vs. 1)
Fluoroscopy time (min)	12.7 ± 9.5	19.8 ± 12.2	21.6 ± 8.9	< 0.001	0.558	< 0.001	< 0.001
Total contrast dose (ml)	163.0 ± 69.0	236.3 ± 116.6	278.7 ± 168.4	< 0.001	0.011	< 0.001	< 0.001
Post-procedural troponin (ng/L)	2.32 ± 5.8	1.82 ± 6.74	2.74 ± 7.24	0.109	1.0	0.272	1.0
Post-procedural creatinine (mg/dl)	1.22 ± 1.20	1.16 ± 0.93	1.19 ± 1.01	0.887	1.0	1.0	1.0
Post-procedural CK-MB (ng/L)	1.4 ± 4.7	4.9 ± 5.5	4.3 ± 4.7	0.375	1.0	0.091	0.087
Procedural cost (euros)	1900 ± 1856	3329 ± 2325	3152 ± 1814	< 0.001	1.0	< 0.001	< 0.001
Total days of hospital stay	6.5 ± 5.9	8.5 ± 6.4	6.0 ± 4.3	< 0.001	< 0.001	< 0.001	1.0
Myocardial injury		43 (17.2)	25 (13.8)		0.141		
Periprocedural MI		5 (2.0)	4 (2.2)		1.0		

**TABLE 4:** Intravascular imaging use.

Lesions (*n* = 1,591)	Group 1 (*n* = 1,137)	Group 2 (*n* = 265)	Group 3 (*n* = 189)	*P*-value (global)	*P*-value (3 vs. 2)	*P*-value (3 vs. 1)	*P*-value (2 vs. 1)
Use of intravascular imaging (%)	3 (0.3)	14 (5.3)	8 (4.2)	< 0.001	1.0	< 0.001	< 0.001

Rate of in-hospital MACE was low and not significantly different between Group 2 and 3 as well the incidence of periprocedural myocardial injury or type 4a MI (Table 3).

### Clinical follow-up

**FIGURE 3:**
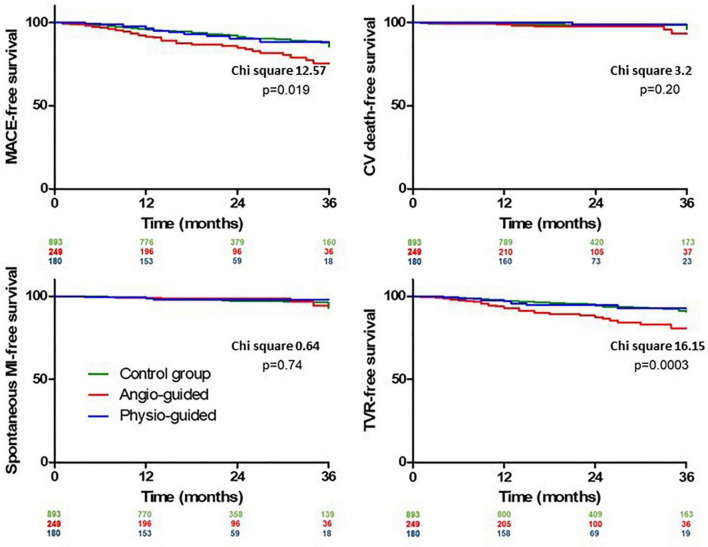
Kaplan Meier curves for out-of-hospital outcomes comparing group 1 (Control Group), group 2 [Angiography-Guided percutaneous coronary intervention (PCI)] and group 3 (Physiology-guided PCI).

**FIGURE 4:**
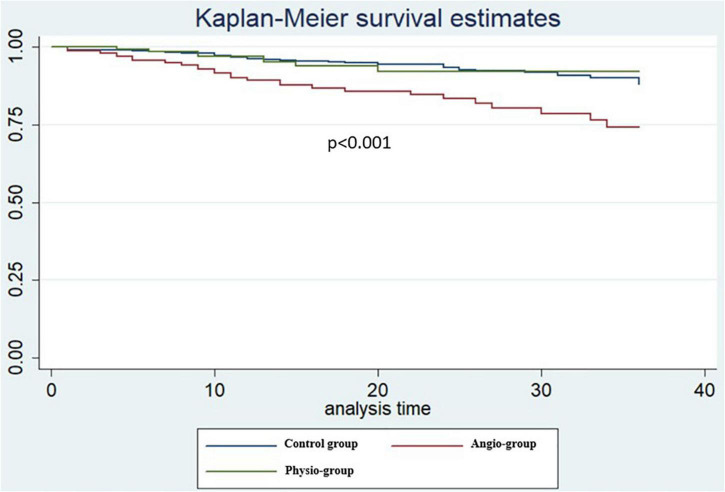
Kaplan Meier curves patients with chronic coronary syndromes only at pairwise log rank comparison [Physiology-guided percutaneous coronary intervention (PCI) vs. Angiography-guided PCI *p* = 0.007, Physiology-guided PCI vs. Control Group *p* = 0.89].

The median length of follow up (FU) for the overall population was 21 months (IQR 14–32). A total of 124 (9.3%) MACEs were observed at 36 months including 33 (2.4%) MI, 84 (6.3%) TVR, and 24 (1.8%) CD. The rate of MACE was higher in Group 2 (14.9%) compared both to Group 1 and 3 (8.2% and 7.2%, *p* < 0.001 and *p* = 0.004) while it was not significantly different between Group 1 vs. 3 (*p* = 0.765). The difference in the primary endpoint was mainly driven by the rate of TVRs that were not significantly different comparing Group 3 to Group 1 (5.0% vs. 5.3%) but significantly higher in Group 2 (11.2%) compared to both Group 1 and 3 (*p* < 0.001 Figure 3; Table 5). Considering the possible interference of clinical presentation on subsequent outcomes, given the higher baseline risk profile of unstable patients, we reiterated survival analysis on patients with chronic coronary syndromes only; results consistently showed a lower incidence of MACE in Physiology-guided PCI, driven by a reduction in TVR (Figure 4; Table 6).

**TABLE 5:** Out-of-hospital outcomes.

Patients (*n* = 1,322)	Group 1 (*n* = 893)	Group 2 (*n* = 249)	Group 3 (*n* = 180)	*P*-value (global)	*P*-value (3 vs. 2)	*P*-value (3 vs. 1)	*P*-value (2 vs. 1)
Follow-up, (months)	25.4 ± 16.2	23.9 ± 15.6	22.0 ± 13.3	0.024	0.650	0.028	0.599
MACE (%)	74 (8.2)	37 (14.9)	13 (7.2)	0.004	0.015	0.765	0.003
Myocardial infarctions	25 (2.8)	5 (2.0)	3 (1.7)	0.580	1.000	0.606	0.655
Cardiac deaths	16 (1.8)	7 (2.8)	1 (0.6)	0.224	0.147	0.334	0.311
TVR	47 (5.3)	28 (11.2)	9 (5.0)	0.002	0.024	1	0.001
cardiac hospitalizations	164 (18.4)	80 (32.1)	48 (26.7)	< 0.001	0.241	0.018	< 0.001
All-cause death	33 (3.7)	19 (7.6)	4 (2.2)	0.048	0.048	0.620	0.030

**TABLE 6:** Out of hospital outcomes for patients with chronic coronary syndromes.

CCS patients (*n* = 969)	Group 1 (*n* = 669)	Group 2 (*n* = 161)	Group 3 (*n* = 139)	*P*-value (global)	*P*-value (3 vs. 2)	*P*-value (3 vs. 1)	*P*-value (2 vs. 1)
Follow-up, (months)	21.9 ± 9.8	20.5 ± 10.8	19.5 ± 9.5	0.013	0.363	0.006	0.100
MACE (%)	46 (6.9)	27 (16.8)	8 (5.8)	< 0.001	0.002	1.000	0.000
Myocardial infarction	13 (1.9)	3 (1.9)	2 (1.4)	0.923	1.000	1.000	1.000
Cardiac death	12 (1.8)	6 (3.7)	0 (0.0)	0.057	0.051	0.462	0.308
Target vessel revascularization	29 (4.3)	21 (13)	7 (5.0)	< 0.001	0.009	1	< 0.001
Cardiac hospitalizations	111 (16.6)	56 (34.8)	33 (23.7)	< 0.001	0.052	0.166	< 0.001

No significant difference was observed in the incidence of MI and CD. Cardiac hospitalizations were significantly higher in Group 2 and 3 compared to Group 1 (*p* < 0.001) without significant differences between Group 2 and 3 (*p* = 0.26). These results were confirmed at lesion-level analysis. Specifically, the difference in TVR between Group 2 and 3 was statistically significant (10.9% vs. 4.8%, *p* = 0.004).

Cox regression analysis confirmed the significant difference in MACE rate among the groups even after adjustment for potential confounders (age, gender, hypertension, diabetes, chronic kidney disease, previous MI, previous PCI, clinical presentation, LVEF, and multivessel disease) with strategy of revascularization and previous MI being the only factors influencing the outcomes (Table 7).

**TABLE 7:** Cox regression analysis for major adverse cardiovascular events (MACE).

	Hazard ratio	95% confidence interval	*p*
Age	1.008	0.990–1.027	0.373
Male gender	0.838	0.569–1.234	0.371
Hypertension	1.267	0.741–2.168	0.387
Diabetes	1.379	0.945–2.014	0.096
Chronic kidney disease	0.714	0.331–1.540	0.390
Previous MI	1.625	1.056–2.500	0.027
Previous PCI	1.166	0.773–1.758	0.464
Clinical presentation			
NSTEMI vs. CCS	1.392	0.681–2.846	0.364
STEMI vs. CCS	1.341	0.889–2.022	0.162
Ejection fraction < 40%	1.855	0.899–3.825	0.094
Multivessel disease	1.414	0.926–2.160	0.109
Revascularization strategy without post-PCI functional assessment	1.893	1.002–3.575	0.049

**FIGURE 5:**
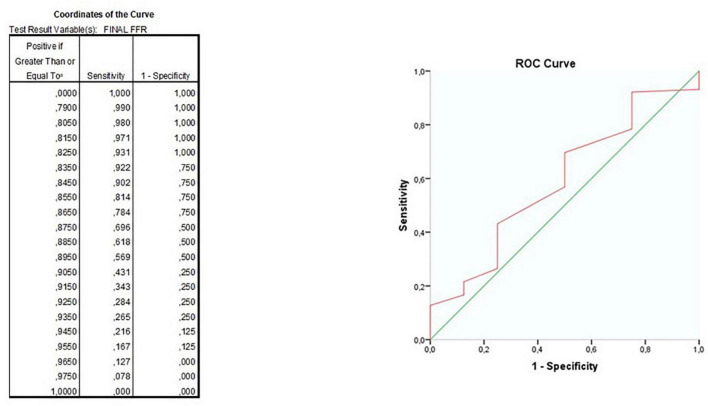
ROC curves for the ability of final fractional flow reserve (FFR) value to predict major adverse cardiovascular events (MACE)-free survival.

Yet, ROC curve analysis (Figure 5) for cases of Physiology-guided PCI in which FFR was the main technique for guiding PCI, showed a modest capacity to predict event free survival with an AUC of 59%; the best cutoff for MACE free survival still appeared to be 0.90 (sensitivity 43%, specificity 75%).

## Discussion

Despite physiological assessment after PCI seems a reasonable strategy to have an immediate measure of the efficacy of revascularization, its use in clinical practice still remains limited. In the present study we present the result of a real-world application of this approach in patients who underwent a physiological assessment of intermediate coronary stenosis.

The main results are the following:

(1) Post PCI IPA is performed in a minority of patients while a persistently suboptimal result is rather frequent in spite of an acceptable angiographic result.

(2) A “Physiology-guided PCI” is a feasible and safe approach to check the immediate result of revascularization.

(3) Physiology-guided PCI was associated to a better outcome as compared to Angiography-guided PCI.

Despite being strongly recommended by current guidelines on revascularization, IPA is underutilized in clinical practice (14). In addition, when ischemic lesions are recognized and treated by PCI, the operators repeat IPA after PCI only in a minority of cases. Our retrospective study confirms these findings, showing that the majority of patients are still treated by an Angiography-guided PCI even after a significant result at IPA. This is quite surprising because physiological reassessment at this stage is obviously costless and associated with a limited increase in procedural time. However, when an IPA is performed after PCI, a suboptimal result is quite frequent despite an angiographically satisfying result (15), including lesions still below the “ischemic” threshold (16, 17). In our study we confirm these findings but, unlike other studies enrolling a wide spectrum of different lesions and patients with an indication to PCI, we focused on intermediate coronary artery stenosis that are the main field of application of IPA and for which a class IA was assigned by ESC Guidelines on myocardial revascularization. In our cohort about 50% of all lesions exhibited a post-PCI suboptimal physiological result (FFR < 0.90), although rarely 0.80. These suboptimal results can be further ameliorated by additional interventions, such as stent post-dilation or stenting proximally or distally to the already implanted stent.

The main novelty of the present study is the evidence of a potential beneficial effect of a Physiology-guided PCI over a standard Angiography-guided approach. Indeed, we found that Physiology-guided PCI resulted in a similar final FFR value and in a comparable rate of MACE in comparison to control patients in whom PCI was deferred based on the IPA of intermediate lesions. Moreover, the rate of MACE was significantly lower than that observed in Angiography-guided PCI in the absence of a relevant increase of procedural complexity and of in-hospital events, mainly driven by a reduction in the rate of TVR. Length of stay of group 2 appeared to be slightly longer but this observation may be mostly attributable to the higher prevalence of ACS patients in this group.

Recently, the TARGET-FFR (18) trial demonstrated that a physiology-guided strategy was associated with a lower rate of suboptimal results in comparison to a standard angiographic approach. However, this appeared to have a marginal effect on clinical events. Notably, despite a suboptimal result occurring in less than 50% of cases, in only two thirds of these lesions investigators were able to identify a substrate for further optimization. This means that not only a suboptimal result is frequent but that it is often hardly amenable for improvement because of the presence of diffuse disease or other technical reasons. In our cohort, only 22% of all post-PCI evaluations were followed by any optimization; this could be due not only to the overall quite high post-PCI results but mostly to the lack of a shared and definite cut-off to consider and optimal. Actually, we confirmed that 0.90 is the cutoff with the higher predictive value but this result was achieved with a very modest sensitivity.

In our study, the inclusion of only intermediate coronary stenosis with an indication to diagnostic IPA has played a role in reducing the prevalence of suboptimal and ischemic result after PCI. In this setting, however, it is even more remarkable the reduction in TVR in comparison to the standard angiography-based approach. In addition, it is interesting to note that this comes not only without a significant increase in the complexity of the procedure, as clearly demonstrated by a similar fluoroscopy time but even without a significant increase in the rate in-hospital events, in the length of stay and in total costs, possibly due to the surrogate use of the pressure wire instead of the expensive intracoronary imaging for PCI guidance and optimization. However, this strategy should be implemented using a quite rigorous algorithm aimed at identifying not only the presence of any suboptimal result, but also if this is due to a diffuse disease or to a geographically missed focal lesion located distally or proximally to the stented segment. In an effort to standardize this evaluation, several groups, including ours (9), have suggested a systematic approach to correctly diagnose and treat a suboptimal functional result.

The first step in any systematic post-PCI IPA is to define what “a suboptimal result” means. During the years, several cut-off values for post-PCI FFR have been proposed although most of the data bulks around 0.89 and 0.91 (19), with recent evidence hinting at the possibility of different cutoff for different vessels (20). In line with previous data, our study suggests that the best cut-off value is above 0.9, but this threshold has, however, a modest ability of predicting MACE occurrence at follow-up.

Accordingly, a large meta-analysis of 105 studies confirmed the relation among FFR below 0.9 and the risk of MACE or TVR and the recent FFR-SEARCH study which showed that patients with post-PCI FFR < 0.9 had 6.2% incidence of TVR at 48 months vs. 3.9% for FFR above 0.9 (18, 21).

Once a “suboptimal functional result” is observed, choosing how to manage the lesion is a complex task; indeed, a suboptimal post-PCI FFR could recognize a wide range of causes from stent under-expansion, to edge dissection, missed stenosis, diffuse atherosclerotic disease etc. One of the limitations of the postprocedural IPA of coronary circulation is its limited ability to identify the causes of a suboptimal result. In this regard, a pressure pullback maneuver can help in the identification of the causes of a suboptimal result limiting intracoronary imaging to doubtful cases (8).

### Limitations

As any other observational study, the PROPHET-FFR study suffers from some limitations. First of all, it is a single center study with a retrospective design. This is clearly demonstrated by the fact that of over 1,300 patients, only 180 received post-PCI physiology and of those, only 12% (22°pts) received post-PCI optimization. However, this design is intentional in the present phase to take a snapshot of the preference of the operators in a center with a large experience in coronary physiology without forcing them to follow a specific algorithm. For example, in the present study the pressure pullback maneuver was not systematically performed, neither in the pre-PCI assessment, when new indexes, such as the pullback pressure gradient (PPG) proposed by Collet et al. (21) could help in planning the procedure, nor in the post-PCI phase. This is a limitation of the study which will be amended in the ongoing prospective phase in which we will test a standardized approach to a fully “Physiology-guided PCI” and compare the outcome results with those obtained in the retrospective phase (8). We believe that the use of this approach could improve clinical outcomes by reducing the number of those lesions with a suboptimal functional result not further optimized that represent, in our study as in other similar studies (16–18), the most important limitation.

## Conclusion

In conclusion, our observational study suggests that “Physiology-guided PCI” in intermediate coronary stenosis is a feasible strategy, associated with a significant cost sparing and more importantly with potential favorable impact on mid-term prognosis. The lack of a strong cutoff and the need for a systematic approach to localize and manage the suboptimal results make imperative the completion of prospective studies using a standardized physiological approach.

## Data availability statement

The raw data supporting the conclusions of this article will be made available by the authors, without undue reservation.

## Ethics statement

The studies involving human participants were reviewed and approved by Comitato Etico del Policlinico Gemelli di Roma. The patients/participants provided their written informed consent to participate in this study.

## Author contributions

AL: conception and design, analysis and interpretation of data, drafting, and revising critically the manuscript for important intellectual content. SM: analysis of data and drafting of the manuscript. GZ, PC, EB, DG, FD, GA, AV, EP, AD, GC, DD’A, RV, RM, AB, ER, and CA: collection, analysis, and interpretation of data. FB, CT, and FC: important intellectual contribution. All authors contributed to the article and approved the submitted version.

## Footnotes

1
https://gise.it/statisticheNazionali

## Abbreviations

CCS: chronic coronary syndrome
CD: cardiac death
cFFR: contrast-FFR
dPR: diastolic resting pressure ratio
FFR: Fractional Flow Reserve
iFR: instantaneous wave free ratio
IPA: invasive physiological assessment
LAD: left anterior descending
LVEF: Left Ventricle Ejection Fraction
MACE: Major Adverse Cardiovascular Events
MI: myocardial infarction
NHPR: non-hyperemic pressure ratio
NSTEACS: non-ST segment elevation acute coronary syndromes
PCI: Percutaneous Coronary Intervention
RFR: resting full-cycle ratio
STEMI: ST segment elevation myocardial infarction
TVR: Target Vessel Revascularization.
